# Effects of Fumaric Acids on Cuprizone Induced Central Nervous System De- and Remyelination in the Mouse

**DOI:** 10.1371/journal.pone.0011769

**Published:** 2010-07-23

**Authors:** Darius Moharregh-Khiabani, Alexander Blank, Thomas Skripuletz, Elvira Miller, Alexandra Kotsiari, Viktoria Gudi, Martin Stangel

**Affiliations:** 1 Department of Neurology, Hannover Medical School, Hannover, Germany; 2 Center for Systems Neuroscience, Hannover, Germany; Julius-Maximilians-Universität Würzburg, Germany

## Abstract

**Background:**

Fumaric acid esters (FAE) are a group of compounds which are currently under investigation as an oral treatment for relapsing-remitting multiple sclerosis. One of the suggested modes of action is the potential of FAE to exert a neuroprotective effect.

**Methodology/Principal Findings:**

We have investigated the impact of monomethylfumarate (MMF) and dimethylfumaric acid (DMF) on de- and remyelination using the toxic cuprizone model where the blood-brain-barrier remains intact and only scattered T-cells and peripheral macrophages are found in the central nervous system (CNS), thus excluding the influence of immunomodulatory effects on peripheral immune cells. FAE showed marginally accelerated remyelination in the corpus callosum compared to controls. However, we found no differences for demyelination and glial reactions *in vivo* and no cytoprotective effect on oligodendroglial cells *in vitro*. In contrast, DMF had a significant inhibitory effect on lipopolysaccharide (LPS) induced nitric oxide burst in microglia and induced apoptosis in peripheral blood mononuclear cells (PBMC).

**Conclusions:**

These results contribute to the understanding of the mechanism of action of fumaric acids. Our data suggest that fumarates have no or only little direct protective effects on oligodendrocytes in this toxic model and may act rather indirectly via the modulation of immune cells.

## Introduction

Multiple sclerosis (MS) is the most common cause of neurological disability in young adults. Its pathology is characterised by an inflammation-mediated demyelination and axonal loss in the central nervous system (CNS) white and grey matter [1]. The current treatment options for relapsing remitting MS (RRMS) are only partially effective and need a parenteral route of administration. Still oral therapeutics, which could either prevent tissue damage and/or support repair mechanisms are not available.

Fumaric acid esters (FAE) can be administered orally and are currently being evaluated for the treatment of MS. A recently published randomised, placebo-controlled phase II study showed significant positive effects of FAE on various MRI parameters in MS patients with relapsing-remitting MS. This study utilised a second generation FAE formulation (BG00012) consisting of dimethylfumaric acid (DMF) as single compound that is thought to represent the active component of FAE [2]. The mode of action is not yet clear, but both immunomodulatory and neuroprotective effects are being discussed [3]. FAE have been demonstrated *in vitro* to exert immunomodulatory effects on T-cells [4], B-cells [5], and dendritic cells [6], [7]. The immunomodulatory properties of FAE were also investigated in the rodent model of myelin oligodendrocyte glycoprotein (MOG) induced experimental autoimmune encephalomyelitis (MOG-EAE), an animal model that mimics several aspects of MS [8]. In mice treated with FAE a significant therapeutic effect on the disease course could be found. Furthermore, the numbers of microglia/macrophages but not of T-cells were reduced in the inflammatory lesions.

Beside its immunomodulatory effects FAE bear the potential for neuroprotective effects via detoxifying pathways. Cell culture experiments with rat mixed glia (microglia and astrocytes) showed an upregulation of the detoxification enzyme NAD(P)H: quinine oxidoreductase (NQO-1), a reduction of the intracellular glutathione content, and a reduction of the neurotoxic agent nitric oxide by FAE treatment [9]. In human peripheral blood mononuclear cells (PBMC) DMF induced an upregulation of the anti stress protein heme oxygenase 1 (HO-1), which led to a reduction of the intracellular glutathione content [10].

To further investigate the cytoprotective potential of FAE we used the toxic cuprizone model of demyelination in mice. The cuprizone model is well established to follow both demyelination and spontaneous remyelination in the CNS white and grey matter [11]–[14].

## Materials and Methods

### Animals

C57BL/6 male mice were obtained from Charles River (Sulzfeld, Germany). Animals underwent routine cage maintenance once a week and were microbiologically monitored according to Federation of European Laboratory Animal Science Associations recommendations [15]. Food and water were available *ad libitum*. All research and animal care procedures were approved by the Review Board of the care for Animals Subjects of the district government (Lower Saxony, Germany) and performed according to international guidelines on the use of laboratory animals.

### Induction of demyelination and treatment with fumarates

Demyelination was induced by feeding 8-week-old male C57BL/6 mice a diet containing 0.2% cuprizone (bis-cyclohexanone oxaldihydrazone; Sigma-Aldrich Inc., St. Louis, MO) mixed into a ground standard rodent chow for 5 weeks. Animals were then put on standard rodent chow without cuprizone to induce remyelination.

Cuprizone treated mice received 15mg monomethylfumarate (MMF) or 15mg DMF per kg body weight (both from Sigma-Aldrich Inc., St. Louis, MO) twice daily as described by Schilling et al. [8]. MMF and DMF were diluted in 200µl 0,08% methocel/H_2_O. Cuprizone mice treated with 200µl 0,08% methocel/H_2_O without fumarates served as controls. For treatment oral gavage was used to ensure exact dosing and to avoid compound degradation. Treatment was administered starting from day 1 of cuprizone feeding until termination.

At different time points (weeks 4, 5, and 6) animals were perfused with 4% paraformaldehyde in phosphate buffer via left cardiac ventricle as previously described [16]. A group size of five to six animals was investigated at all time points. The brains were removed, postfixed in 4% paraformaldehyde and paraffin embedded. For light microscopy, 7µm serial paraffin sections were cut and dried at 37°C overnight.

### Histology and immunohistochemistry

Histology and immunohistochemistry were performed as previously described [13]. Briefly, 7µm serial sections between bregma −0.82 and bregma −1.8 (according to mouse atlas by Paxinos and Franklin, [17] were analysed. Sections were stained for myelin with Luxol-fast-blue periodic acid-Schiff base (LFB-PAS) (Sigma-Aldrich Inc., St. Louis, MO). For immunohistochemistry, paraffin-embedded sections were dewaxed, rehydrated, and boiled for 5 minutes in 10mmol/L citrate buffer (pH 6.0). Sections were quenched with H_2_O_2_, blocked for 1 hour in phosphate-buffered saline (PBS) containing 3% normal goat serum, 0.1% TritonX-100, and then incubated overnight with primary antibody. The following primary antibodies were used: For myelin proteins proteolipid protein (PLP) (mouse IgG, Serotec, Düsseldorf, Germany), myelin basic protein (MBP) (mouse IgG, Sternberger Monoclonals Inc., Berkeley, CA), myelin oligodendrocyte glycoprotein (MOG) (mouse IgG, 1∶2 hybridoma supernatant, gift from C. Linington), for astrocytes glial fibrillary acidic protein (GFAP) (mouse IgG, Chemicon, Hampshire, UK), for oligodendrocytes Nogo-A (rabbit IgG, Chemicon, Hampshire, UK), for oligodendrocyte precursor cells (OPC) Olig-2 (rabbit IgG, Abcam, Cambridge MA), for activated microglia Mac-3 (rat IgG, BD Pharmingen, Germany), and for axonal damage Alzheimer precursor protein (APP) (mouse IgG, 1∶800 Chemicon). After washing, sections were further incubated with biotinylated secondary antibody (Vector Laboratories, Burlingame, UK) for 1hour, followed by peroxidise-coupled avidin-biotin complex (ABC Kid, Vector Laboratories). Reactivity was visualised with diamino-3,3′benzidine (Dako Cytomation, Hamburg, Germany). Isotype controls and staining without primary antibody were used as controls. There was no significant non-specific staining of mouse immunoglobulin in mouse tissue.

### Determination of de- and remyelination in white and grey matter

To detect de- and remyelination in the corpus callosum myelin stained sections for LFB, MBP, MOG, and PLP were scored by three blinded observers using a scale from 0 (complete demyelination) to 3 (normal myelin). Cortical myelin was scored using a scale from 0 (complete demyelination) to 4 (normal myelin). Scoring of myelination was done at the light microscope (Olympus, Hamburg, Germany) as previously described [12], [13].

### Quantification of glial reaction

Cells positive for Nogo-A, Olig-2, GFAP, and Mac-3 with identified nucleus (counterstained with haematoxylin) were counted left and right of the midline within the corpus callosum within an area of at least 0.125mm^2^ using a magnification of ×40 (Olympus, Hamburg, Germany). For axonal damage Alzheimer precursor protein (APP) positive ovoids were counted as described above. The results were expressed as cell number per mm^2^.

### Mixed glial cell cultures

Primary cultures of mixed glial cells were prepared from neonatal Sprague–Dawley rat cerebra as described [18]. Brains were freed from meninges under a dissecting microscope, dissociated mechanically and enzymatically with trypsin and DNAse (both from Sigma-Aldrich Inc., St. Louis, MO). Finally, a single cell suspension was generated by shearing through a glass Pasteur pipette with flamed tip. Cells were cultured in DMEM including glutamine (Gibco, Karlsruhe, Germany) supplemented with 10% heat inactivated fetal calf serum (FCS, Biochrom, Berlin, Germany), 50 U/ml penicillin, and 50 µg/ml streptomycin (Sigma-Aldrich Inc., St. Louis, MO). After several days a confluent astrocytic monolayer developed with both microglia and OPC on top. Cells were cultured for 7 to 10 days before subjected to cytotoxicity assays.

### Oligodendroglial cell line

CG4 cells [19] were kept in a proliferative state in DMEM containing 30% medium conditioned by the neuroblastoma cell line B104 (B104CM) [20], 1% ITS+tissue culture supplement (Becton Dickinson, Bedford, MA., USA), biotin, putrescine, progesterone and antibiotics in poly-L-lysine coated flasks.

### Cytotoxicity assay

Potential cytoprotective effects of DMF and MMF were measured in the oligodendroglial cell line CG4 [19]. Oligodendroglial cell death was induced by the toxic substances sodium nitroprusside (SNP) or H_2_O_2_. For analyses 10^4^ cells were seeded in a poly-L-lysine coated 96 well plate (Nunc, Wiesbaden, Germany) and were pre-treated 24h with different MMF and DMF concentrations (5, 10, 50µM/ml). Then the toxic agent (1 mM SNP, or 0.09mM H_2_O_2_,) was added for another 24h. At the end of the incubation period medium was exchanged by 100µl of medium containing 10% Alamar-Blue® (TRINOVA Biochem GmbH, Gieβen, Germany). After 4 to 6h the colour change of the metabolised AlamarBlue® was measured by the optical density (OD) at 620nm using an ELISA reader (SLT Spectra, SLT Labinstruments GmbH, Crailsheim, Germany). The reduction of AlamarBlue® is an indirect measurement of cell numbers and produces linear results with a high specificity and sensitivity [21], [22]. Cell numbers for each well were calculated by linear regression in relation to a standard curve derived from a daily control of untreated cells plated at different densities [22], [23].

### Nitric oxide production

The influence of DMF and MMF on nitric oxide production of microglia was tested. Nitrite, the stable end product of nitric oxide (NO), was measured with the Griess reaction [24]. For analyses 10^4^ microglia were seeded in a 96 well plate (Nunc, Wiesbaden, Germany) and were pre-treated 24h with different MMF and DMF concentrations (5, 10, 50µM/ml). Then 10ng/ml lipopolysaccharide (LPS) was added for another 24h. For the measurements microglia cell culture supernatant (100µl) was added to an equal volume of Griess reagent (0.1% naphtylethylene-diaminedihydrochloride, 1% sulfanilamide in 2.5% H_3_PO_4_, all from Sigma). The reaction was allowed to proceed for 15min at room temperature before the OD at 540nm was measured in an ELISA reader (SLT Spectra, SLT Labinstruments GmbH, Crailsheim, Germany). The concentration of nitrite was determined by linear regression from a standard curve using known concentrations of sodium nitrite (Sigma-Aldrich Inc., St. Louis, MO) in culture medium.

### Induction of apoptosis of mouse PBMC

To measure the effect of MMF and DMF on PBMC, blood from mice killed by cervical dislocation was collected by heart puncture into EDTA vacutainer tubes (Sarstedt, Newton, NC). Blood was diluted 1/1 with Dulbecco's PBS. PBMC were separated by density gradient centrifugation using Biocoll (Ficoll®) separating solution (Biochrom, Berlin, Germany). The PBMC layer was collected and washed twice with Dulbecco's PBS and then resuspended at a concentration of 1×10^6^ cells/ml in DMEM (GIBCO 41966) with 10% heat-inactivated FBS (Biochrom, Berlin, Germany) and 1% penicillin-streptomycin (Sigma-Aldrich Inc., St. Louis, MO). 3×10^5^ cells were plated in a Flat Bottom 24-Well plate (Sarstedt, Newton, NC), and treated with MMF and DMF in different concentrations (10, 20, and 50µM/ml). 10µl methanol was used as solvent control. PBMC without any addition of MMF or DMF or methanol served as a medium control. Cells were incubated for 48h. Cell death and apoptotic cells were assessed by using FITC Annexin V Apoptosis Detection Kit I (BD Pharmingen, Germany). After 20min incubation with FITC Annexin V (1∶100) and PI (1∶100) dead cells and apoptotoc cells were determined by FACS analysis on a FACScalibur Becton-Dickinson flow cytometer.

### Statistical analysis

Statistical analysis was performed using one-way analysis of variance (ANOVA) followed by the Fisher-protected least-significant difference test for post hoc comparison if appropriate. All data are given as arithmetic means ± standard error of the mean (SEM). *P* values of the different ANOVAs are given in the Results, while group comparisons derived from post hoc analysis are provided in the figures. In the latter case, significant effects are indicated by asterisks (*P<0.05; **P<0.01; ***P<0.001).

## Results

### MMF and DMF have only minor effects on remyelination in the corpus callosum

**Figure 1:**
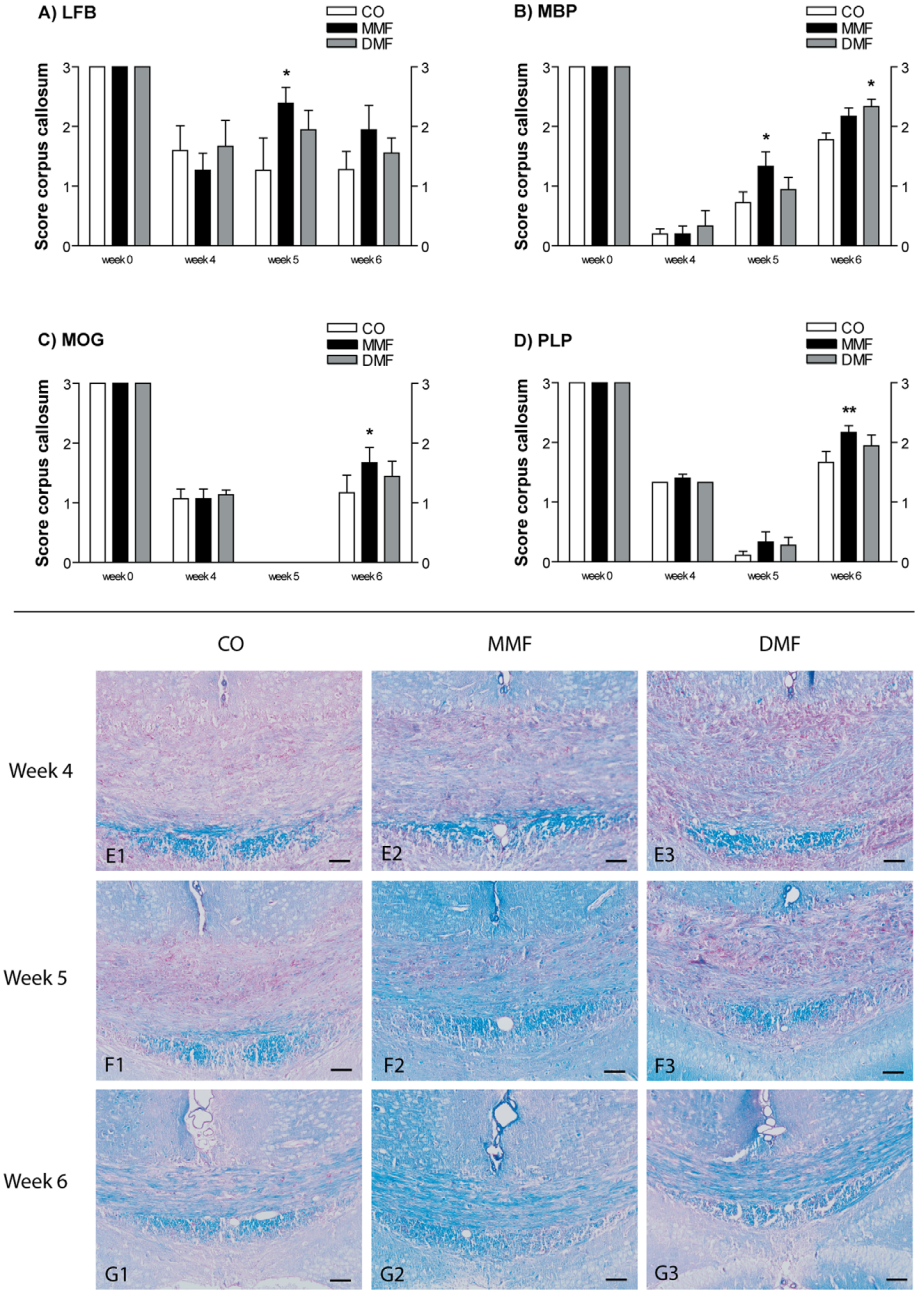
Influence of DMF and MMF on de- and remyelination in the corpus callosum. Myelination in the corpus callosum was demonstrated by scoring of LFB-PAS stained sections (A) and the myelin proteins MBP (B), MOG (C), and PLP (D). In the corpus callosum score of 0 represents complete loss of myelin while score of 3 represents normal amount of myelin. Results are shown as means ± SEM. Significant post hoc effects versus controls (CO) are indicated by asterisks (*p<0.05, **p<0.01). In E1-G3 representative images of LFB-PAS stained sections of the corpus callosum are shown. Scale bars: 100 µm.

To evaluate the sequential loss and re-expression of myelin the histochemical LFB staining and immunohistochemical myelin protein stainings for MBP, PLP, and MOG were used. At week 4 there were no differences in the myelin scores for all investigated myelin proteins and LFB staining. At week 5, when remyelination in the corpus callosum starts, there was a small but significant increase of LFB staining intensity in MMF treated animals (one-way ANOVA p = 0.01, Fig. 1A, A1–C3). In DMF treated mice this increase was not significant. At week 6 nearly the same amount of myelin was seen in the corpus callosum indicating that remyelination remained on the same level. As described previously, the dynamics of myelin protein expression differed between myelin proteins in the corpus callosum [25]. Maximal degradation of MBP was detectable after 4 weeks of cuprizone exposure. At week 5 of cuprizone feeding re-expression of MBP was evident and further increased in week 6. Treatment with MMF resulted in significantly higher MBP expression after 5 weeks of cuprizone exposure, while DMF treated mice showed higher MBP expression in week 6 (one-way ANOVA p<0.0001, Fig. 1B). For PLP and MOG maximal degradation was detectable after 5 weeks of cuprizone feeding. In week 6 MMF treated mice showed higher re-expression of MOG (one-way ANOVA p<0.0001, Fig. 1C) and PLP (one-way ANOVA p<0.0001, Fig. 1D).

**Figure 2:**
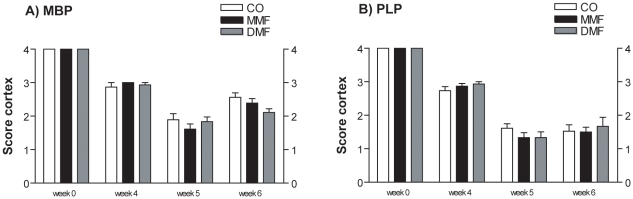
Influence of DMF and MMF on de- and remyelination in the cortex. Cortical myelin was demonstrated by scoring of MBP (A) and PLP (B). In the cortex score of 0 represents complete myelin protein loss, score of 4 represents normal myelin protein amount. Results are shown as means ± SEM.

To determine cortical myelination, PLP and MBP stained section were analyzed. LFB and MOG stained brain sections were not investigated, because of their low sensitivity to demonstrate cortical myelin [12]. In the cortex, both MBP and PLP expression showed no significant differences for MMF and DMF treated mice at the time points investigated (Fig. 2A–B).

### MMF and DMF are not protective for OPC, oligodendrocytes, and axons

**Figure 3:**
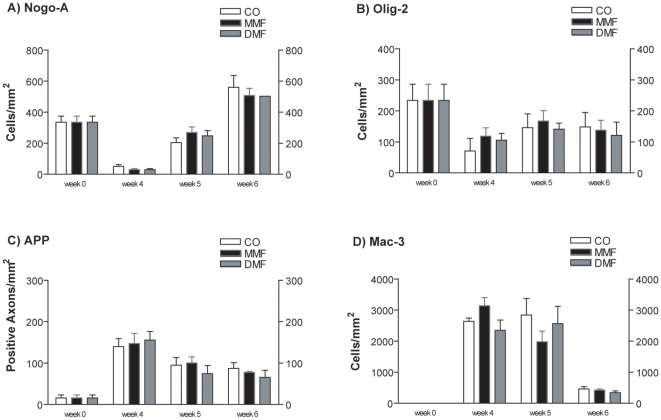
Influence of DMF and MMF on glial reactions during de- and remyelination. Graphs represent cell numbers of Nogo-A (A), Olig-2 (B), and Mac-3 (D) positive cells in the corpus callosum. In C results of the APP staining to demonstrate axonal damage during cuprizone treatment are shown. Cell numbers are given as cells/mm^2^. Results are shown as means ± SEM.

OPC were visualised using immunohistochemical staining for Olig-2, while mature oligodendrocytes were demonstrated by Nogo-A staining [26]. The density of Olig-2 and Nogo-A positive cells in the corpus callosum was not significantly changed in animals treated with MMF or DMF compared to control mice (Fig. 3A–B).

In the demyelinated corpus callosum after 6 weeks of cuprizone feeding the axonal damage is only minor [27]. Here, axonal injury, as detected by APP immunostaining, was not significantly changed in MMF or DMF treated animals as compared to control mice (Fig. 3C).

### Fumarates do not influence microgliosis and astrocytosis in the cuprizone model

In order to study activated microglia during cuprizone treatment we used the marker Mac-3 [28]. Treatment with MMF and DMF showed no difference in microgliosis in the corpus callosum (Fig. 3D). The extent of astrocytosis that occurs during cuprizone treatment in the corpus callosum was not affected by either MMF or DMF (data not shown).

### Fumarates do not protect oligodendroglial cells from oxidative injury in vitro

**Figure 4:**
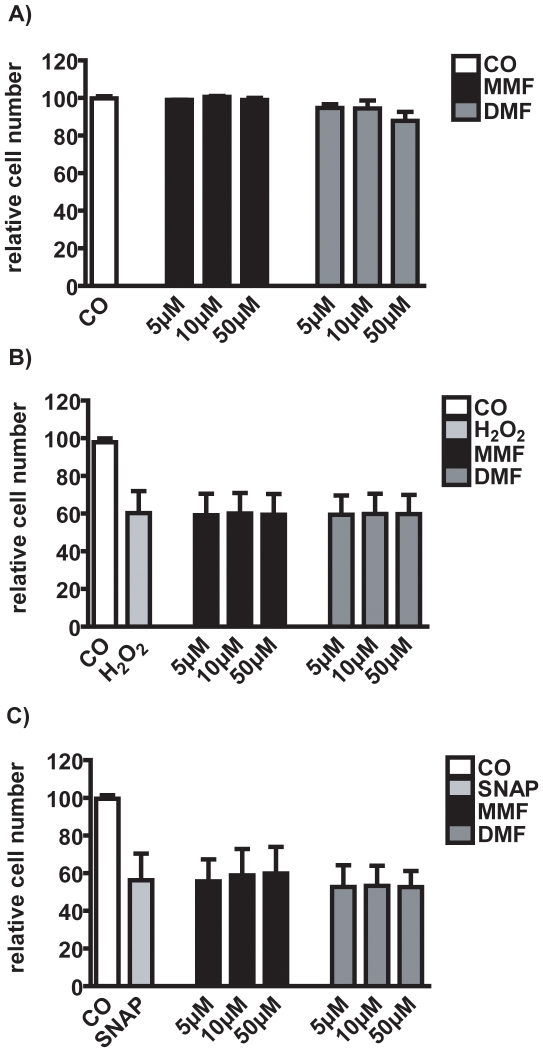
Analysis of cytoprotetive properties of MMF and DMF on the CG4 cell line *in vitro*. CG4 cells were incubated with various concentrations (5, 10, 50µM/ml) of MMF and DMF (A). Cytotoxicity was induced with 0.09mM H_2_O_2_ for 24h (B) and 1mM SNP as NO donor (C). Cell toxicity was measured after 24h using AlamarBlue®. All data represent relative cell numbers to the daily untreated control from three independent experiments ± SEM. CO: medium control.

To further investigate possible cytoprotective properties of MMF and DMF, we used an *in vitro* model that utilised the toxic substances H_2_O_2_ as oxygen radical donor and SNP as nitric oxide (NO) donor on CG4 OPC. MMF and DMF had no significant protective effect on both toxic injuries, suggesting that there is no direct cytoprotective effect of fumarates on OPC (Fig. 4A–C).

### DMF reduce the nitric oxide burst in microglia in vitro

**Figure 5:**
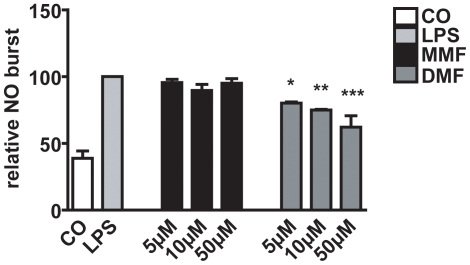
Influence of DMF and MMF on NO production of LPS stimulated microglia. After stimulation for 24 h with 10 ng/ml LPS a dose dependent decrease of NO production in cells incubated with DMF (5, 10, 50µM/ml) could be observed. All data represent three independent experiments compared to a daily standard curve using known concentrations of sodium nitrite in culture medium. Results are shown as means ± SEM. Significant post hoc effects versus controls (treated with LPS) are indicated by asterisks (*p<0.05, **p<0.01, ***p<0.001). CO: medium control without LPS.

In order to study the effects of MMF and DMF on NO burst in microglia we used primary rat microglia *in vitro*. After LPS induced NO burst there was no change after MMF treatment (Fig. 4). In contrast, DMF significatly reduced the amount of nitrite production in a dose dependent manner (one-way ANOVA p<0.0001, Fig. 5).

### DMF but not MMF induces dose dependently cell death in mouse PBMC

**Figure 6:**
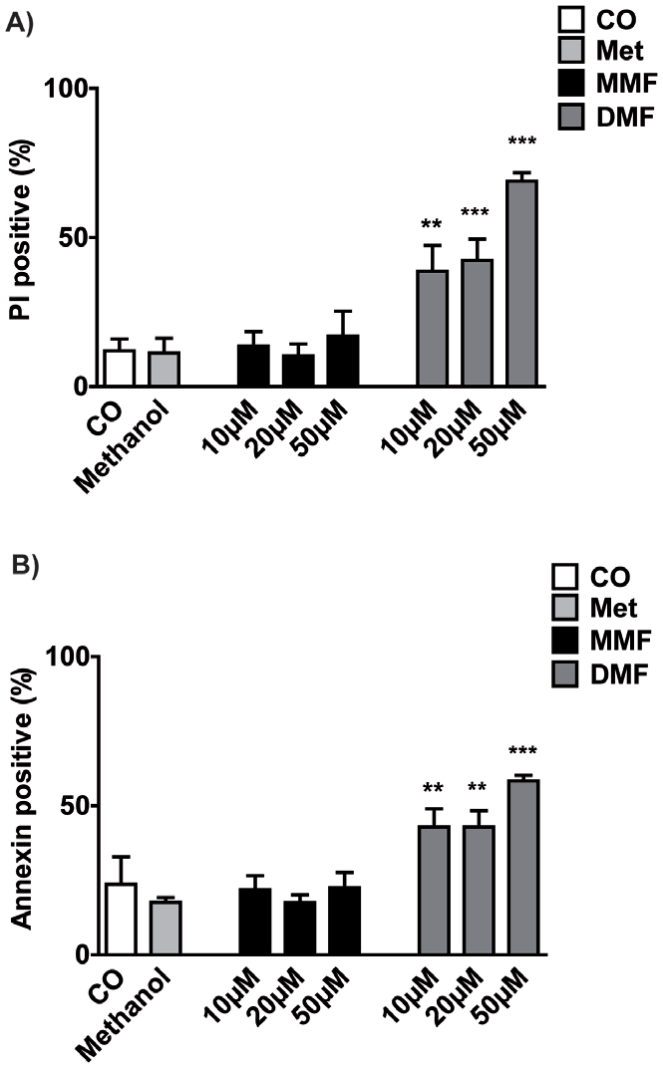
Induction of apoptosis in mouse PBMC by DMF or MMF for 48h as determined with PI (A) and Annexin (B) staining by flow cytometry. For controls PBMC were treated with 1.0% methanol or with medium alone (CO). Results are shown as means ± SEM. Significant post hoc effects versus controls (treated with methanol) are indicated by asterisks (**p<0.01, ***p<0.001).

Since fumarates are known to induce apoptosis in human T-cells [4] we investigated the effects of MMF and DMF on mouse PBMC. As shown in Fig. 6A and B different MMF doses did not induce cell death as measured for PI and annexin positive cells. In contrast, DMF treatment increased the numbers of PI positive as well as annexin positive cells in a dose dependent manner (for both one-way ANOVA p<0.0001).

## Discussion

Preservation from demyelination and enhancement of remyelination are important aims in multiple sclerosis research. Recently, beneficial effects of FAE could be evaluated in MOG-EAE studies in mice as well as in a phase II study in MS patients [2], [8]. Still the mode of action of FAE is not clear and both immunomodulatory and neuroprotective factors have been hypothesised. In this study, the potential of FAE to reduce demyelination and/or to enhance remyelination was investigated in the toxic cuprizone model. The cuprizone model is widely used to study experimental de- and remyelination in mice [11], [29]. Since no breakdown of the blood-brain-barrier (BBB) occurs [30], [31], the cuprizone model offers the opportunity to investigate de- and remyelination without influence of the periphery. Feeding mice 5 weeks with cuprizone induces nearly complete demyelination in the white matter of the corpus callosum. After withdrawal of the toxin spontaneous remyelination occurs [25]. In the current study, mice treated with MMF exhibited showed no difference in myelin protein expression during demyelination in the corpus callosum after 5 weeks of cuprizone treatment. At the end of this period there was a slightly accelerated remyelination in MMF treated animals when assessed by LFB and MBP stainings. This was also evident one week after cessation of cuprizone feeding with slightly higher expression of MOG and PLP in MMF treated mice. Since cortical myelin loss is also a prominent feature in this model [12], de- and remyelination in the cerebral cortex were also investigated. However, DMF and MMF treatment did not significantly change cortical de- and remyelination.

Preceding demyelination, severe depletion of mature oligodendrocytes was observed in the corpus callosum after 4 weeks of cuprizone feeding, while increasing again by weeks 5 and 6. This is in accordance with findings on oligodendrocyte loss in cuprizone induced demyelination [32], [33]. However, no protective effect on oligodendrocytes could be observed in animals treated with FAE. Since cuprizone feeding decelerates the differentiation of oligodendrocytes *in vitro* [34], the impact of FAE treatment on OPC numbers during de- and remyelination was also investigated. However, FAE had no effect on OPC numbers in our *in vivo* model. Along with demyelination microgliosis and astrogliosis occurred in the corpus callosum. Again, no significant differences were observed in FAE treated animals.

Because in the cuprizone model no breakdown of the BBB occurs, blood macrophages and T-cells do not enter the CNS. In contrast to our *in vivo* results, in EAE experiments fumarates led to a reduced Mac-3 positive microglia/macrophage inflammation in the spinal cord as well as a significantly therapeutic effect on the disease course [8]. The differences observed in the EAE model and the cuprizone model may be due to the influence of fumarates on peripheral immune cells. Since both T-cells and peripheral macrophages infiltrate the lesions in EAE this is not the case in the cuprizone model. This is supported by our *in vitro* results where fumarates had no cytoprotective effect on oligodendroglial cells but influenced apoptosis of PBMC. Furthermore, DMF reduced the NO burst in microglia which may indirectly reduce demyelination. These results suggest that the beneficial effects of fumaric acids in inflammatory CNS diseases is rather mediated by the modulation of peripheral immune cells and has only little protective effects on myelin integrity and oligodendrocytes. Since there is only little axonal damage in cuprizone induced demyelination (as compared to the EAE model) this model may not be optimal for the investigation of a direct neuroprotective effect on axons and neurons. Finally, the failure of fumarates to enhance remyelination may also be due to the extremely strong and successful spontaneous remyelination in the cuprizone model. Re-expression of myelin proteins was nearly complete after already one week of remyelination (see Fig. 1) and thus a small effect to accelerate remyelination may not be evident.

In conclusion, we could demonstrate that FAE have no influence on demyelination and only minor effects on remyelination in cuprizone induced myelin loss. The effect of FAE on apoptosis of peripheral T-cells rather suggests that a major contributor to the beneficial effects of FAE observed in EAE and MS may be due to the modulation of peripheral immune cells that cross the BBB.
